# Ethyl acetate extract of *Kaempferia parviflora* inhibits *Helicobacter pylori*-associated mammalian cell inflammation by regulating proinflammatory cytokine expression and leukocyte chemotaxis

**DOI:** 10.1186/s12906-020-02927-2

**Published:** 2020-04-22

**Authors:** Variya Nemidkanam, Yuko Kato, Tetsuo Kubota, Nuntaree Chaichanawongsaroj

**Affiliations:** 1 Program of Molecular Sciences in Medical Microbiology and Immunology, Department of Transfusion Medicine and Clinical Microbiology, Faculty of Allied Health Sciences, Chulalongkorn University, Pathumwan, Bangkok, Thailand; 2 Department of Microbiology and Immunology, Tokyo Medical and Dental University Graduate School of Health Care Sciences, Tokyo, Japan; 3 Research Unit of Innovative Diagnosis of Antimicrobial Resistance, Department of Transfusion Medicine and Clinical Microbiology, Faculty of Allied Health Sciences, Chulalongkorn University, Pathumwan, Bangkok, Thailand

**Keywords:** *Kaempferia parviflora*, *Helicobacter pylori*, Inflammation, Interleukin-8, Chemotaxis

## Abstract

**Background:**

*Kaempferia parviflora* (KP) has been used in traditional Thai medicine to cure gastrointestinal disorders since ancient times. *Helicobacter pylori* is an initiating factor in gastric pathogenesis via activation of massive inflammation, the cumulative effect of which leads to gastric disease progression, including gastric carcinogenesis. Accordingly, the effect of a crude ethyl acetate extract of KP (CEAE-KP) on proinflammatory cytokine production and cell chemotaxis was the focus of this study.

**Methods:**

The cytotoxicity of CEAE-KP (8–128 μg/ml) on AGS (gastric adenocarcinoma) cells was determined at 6, 12 and 24 h using an MTT assay. The effect of CEAE-KP on *H. pylori*-induced interleukin (IL)-8 production by AGS cells was evaluated by ELISA and RT-PCR. The effect of CEAE-KP on monocyte and neutrophil chemotaxis to *H. pylori* soluble protein (sHP) and IL-8, respectively, was determined using a Boyden chamber assay with THP-1 or HL-60 cells.

**Results:**

CEAE-KP reduced AGS cell viability in a concentration- and time-dependent manner, but at 8–16 μg/ml, it was not cytotoxic after 6–24 h of exposure. Coculture of AGS cells with CEAE-KP at a noncytotoxic concentration of 16 μg/ml and *H. pylori* reduced IL-8 secretion by ~ 60% at 12 h, which was consistent with the decreased level of mRNA expression, and inhibited neutrophil chemotaxis to IL-8. sHP (100 ng/ml) induced marked monocyte chemoattraction, and this was decreased by ~ 60% by CEAE-KP.

**Conclusion:**

CEAE-KP might serve as a potent alternative medicine to ameliorate the inflammation mediated by *H. pylori* infection.

## Background

*Helicobacter pylori* is of considerable concern as a gastric pathogen and is one of the factors associated with peptic ulcers and various types of gastric cancer, such as gastric adenocarcinoma and gastric mucosa-associated lymphoid tissue lymphoma [1]. Approximately half of the population worldwide are infected with *H. pylori* and thus have an increased gastric cancer risk. The common *H. pylori* transmission route is gastro-oral through consuming contaminated food and drink. Eating traditional raw foods, such as pickled fish and papaya salad, has a high predictive value for *H. pylori* infection [2]. Milk from livestock and bottled mineral water have also been found to have *H. pylori* contamination and could play a role in spreading the infection [3, 4]. Because of the effortless route of *H. pylori* transmission, a high infection prevalence is found, especially in countries that have unhygienic food preparation and consumption habits [5, 6]. From 2000 to 2015, clarithromycin and levofloxacin resistance increased in the Asia-Pacific region [7], reducing the efficacy of *H. pylori* eradication regimens. Unfortunately, the prevention of *H. pylori* infection by establishing a vaccine is still unsuccessful due to *H. pylori* antigen adaptation and difficulties in vaccine delivery [8]. As a result, various medicinal plants have been explored for their antimicrobial and pharmacological activities to combat *H. pylori* infection with minimal side effects.

*H. pylori* alters host gastric cell signaling pathways via secreted virulence factors, including upregulating inflammation. The bacteria express a variety of soluble protein antigens that initiate an inflammatory signal, such as cytotoxin-associated gene A (Cag A), vacuolated toxin A (Vac A), neutrophil-activating protein and heat shock protein [9]. Soluble *H. pylori* proteins (sHPs) stimulate eosinophil, monocyte and neutrophil functions, leading to the production of related inflammatory cytokines and reactive oxygen species [10–12]. Most of the antigens activate NF-κB functions through the MAPK/ERK and PI3K/Akt signaling pathways [13, 14] and result in the extended production of proinflammatory molecules, including interleukin-8 (IL-8), interleukin-1β (IL-1β), and monocyte chemoattractant protein-1 (MCP-1) [15]. These molecules function as chemokines to recruit neutrophils and monocytes into the infection area. Consequently, the functions of inflammatory cells enhance the severity of gastric disease [16–18]. Incurable *H. pylori* infection can lead to chronic gastritis, which is an initial stage of gastric carcinogenesis. Recently, evaluation of the inflammation-related carcinogenesis mechanism revealed that inflammatory byproducts induce DNA damage that then leads to mutation and genomic instability of the cell [19].

*Kaempferia parviflora* Wall. Ex Baker (KP), or Kra-Chai-Dum, is a Thai medicinal plant within the ginger (*Zingiberaceae*) family. Its rhizome has traditionally been used to relieve gastric symptoms. Methoxyflavone was identified as an active component in the KP rhizome with a variety of biological properties, while KP activities include the support of sexual performance and antioxidant, antitumor growth and metastasis, bactericidal and anti-inflammatory effects [20–24]. The ethanol extract of KP inhibits *Cronobacter* spp., enterohemorrhagic *Escherichia coli* and *H. pylori* growth, which are offensive gastro-enteric pathogens [25]. In a previous study, the crude ethyl acetate extract of KP (CEAE-KP) was shown to reduce *H. pylori* pathogenic function by inhibiting their internalization process in human laryngeal carcinoma cells [26]. The ethanol extract of KP suppressed proinflammatory cytokine gene expression in antigen-stimulated rat basophilic leukemia cells and reduced nitric oxide production in LPS-activated mouse macrophage cells [27]. Moreover, KP has a gastroprotective effect by preventing gastric ulcer formation and maintaining the gastric wall mucus content in a rat model [28].

Because *H. pylori* is a potent stimulant of inflammation-induced cancer and KP might be used as a novel alternative medicine to improve the efficacy of *H. pylori* treatment, we assessed the in vitro regulatory role of CEAE-KP on *H. pylori*-induced inflammation in a human gastric cell line and chemotaxis of relevant inflammatory cells.

## Methods

### Chemicals and reagents

Dimethyl sulfoxide (DMSO) was purchased from Sigma-Aldrich (St. Louis, MO, USA). Thiazolyl blue tetrazolium bromide (MTT) was obtained from Bio Basic (Markham, ON, Canada). Fetal bovine serum (FBS), RPMI, antibiotics and antimycotic agents were procured from GE Healthcare (Chicago, IL, USA). The human IL-8 ELISA kit was purchased from Abcam (Cambridge, UK). The GENEzol reagent kit was obtained from Geneaid Biotech (New Taipei City, Taiwan). RevertAid M-MuLV reverse transcriptase was purchased from Thermo Fisher Scientific (Waltham, MA, USA), and Taq DNA Polymerase was obtained from New England Biolabs (Ipswich, MA, USA).

### Plant materials and extraction

KP was purchased from a traditional Thai drug store in Bangkok, Thailand. The plant material was identified by Dr. Eakarin Saifah of the Department of Pharmaceutical Botany, Faculty of Pharmaceutical Sciences, Chulalongkorn University, Bangkok, Thailand, and a specimen voucher (number ES280306) was deposited at the herbarium of this university. The extraction was performed by the maceration method as described previously [26]. Briefly, roughly ground, air-dried KP was extracted in 200 ml ethyl acetate solvent for 48 h at room temperature. The solvent phase was collected and evaporated at 40 °C until dry to yield the CEAE-KP and weighed. This was then dissolved in DMSO to a concentration of 0.1 g/ml and sterilized by 0.2 μm pore size filtration to form the stock solution, which was stored at − 20 °C in the dark until use.

### Cell lines (AGS, THP-1 and HL-60) and culture

The AGS cell line (kindly provided by Dr. Panan Ratthawongjirakul of the Faculty of Allied Health Sciences, Chulalongkorn University, Thailand) was cultured in complete medium (CM; RPMI 1640 containing 10% (v/v) fetal bovine serum) supplemented with 1% (v/v) antibiotics and antimycotics at 37 °C in 5% CO_2_ and 80% humidity. The cells were harvested at 80–90% cell confluence by 0.25% EDTA-trypsin. The THP-1 cell line was provided by Dr. Tetsuo Kubota (Tokyo Medical and Dental University, Tokyo, Japan). Human promyelocytic leukemia HL-60 cells were obtained from the RIKEN BioResource Center (Tsukuba, Japan). Cells were cultured in CM in the same manner as AGS cells.

### Cell viability (cytotoxicity assay) assessment using thiazolyl blue tetrazolium bromide (MTT)

AGS cells (1 × 10^4^ cells/well) were seeded in 96-well plates and incubated in CM without antibiotics for 24 h. The cells were then washed, and various concentrations of CEAE-KP (8–128 μg/ml) were added to each test well and incubated for 6, 12 and 24 h before 5 mg/ml MTT was added and incubated for an additional 4 h in the dark. The media were then removed, and the cells were permeabilized in 100 μl 10% (w/v) SDS in 0.01 N HCl to solubilize the formazan crystals. The absorbance was read at 570 nm (A_570_) using a Synergy 2 microplate reader (BioTek, Winooski, VT, USA), and the % cell survival was compared with the negative control as follows:

Cell viability (%) = [(A_570(KP extract treated sample)_/A_570(negative control)_)] × 100.

### Bacterial stain and culture conditions

*H. pylori* ATCC 43504 was cultured on 7% (v/v) sheep blood-enriched brain heart infusion agar and incubated at 37 °C for 3 d in an anaerobic jar with an Anaero Pack-MicroAero Gas Pack (Mitsubishi gas chemical, Japan) to create microaerobic conditions (6–12% O_2_ and 5–8% CO_2_).

### Soluble *H. pylori* protein (sHP) extraction

A 3-d-old culture of *H. pylori* was harvested, washed and resuspended in phosphate buffered saline (PBS) at 1 × 10^8^ colony forming units/ml and then lysed by Vibra Cell sonicator (Sonics & Materials, Newtown, CT, USA) with 30 times, 10-s pulses on ice at an amplitude of 40 μm. Following sonication, the residual intact cells and cell debris were removed by centrifugation at 15,000 g for 30 min, and the supernatant was filtered through a 0.2 μm filter to yield the sHP, which was stored at − 80 °C until use. The protein concentration of sHP was determined by the Bradford method using bovine serum albumin as the standard.

### Measurement of IL-8

AGS cells (5 × 10^4^ cells/well) in CM (without antibiotic) were seeded in 24-well plates and incubated for 24 h. Experimental groups were inoculated with *H. pylori* (multiplicity of infection = 1:100) and 16 or 32 μg/ml CEAE-KP and then cultured for 6, 12 or 24 h, as in the MTT assay. The plates were then centrifuged to pellet the cells and debris, and the supernatant was collected. The IL-8 concentration was then determined using a human IL-8 ELISA kit according to the manufacturer’s instructions, reading the absorbance at 450 nm with a microplate reader and calculating the IL-8 concentration from the IL-8 standard curve.

### RNA preparation and reverse transcription polymerase chain reaction (RT-PCR)

AGS cells (3 × 10^5^ cells/well) in CM (without antibiotics) were seeded in 6-well plates and incubated for 24 h. Experimental groups were treated with *H. pylori* at a multiplicity of infection of 1:100 or sHP at 100 ng/ml and were cocultured with 16 or 32 μg/ml CEAE-KP for 12 h. Total RNA was then extracted using a GENEzol reagent kit and converted into cDNA using a reverse transcription system with Oligo (dT)_18_ and RevertAid M-MuLV reverse transcriptase. The cDNA was separately amplified with each primer pair shown in Table 1. Each 25 μl reaction contained 1× PCR buffer, 0.2 mM dNTPs, 0.2 μM each primer, and 0.25 U Taq DNA polymerase. Amplification consisted of 94 °C for 3 min, 35 cycles of 94 °C for 30 s, 57 °C for 30 s, and 72 °C for 30 s, and a final extension at 72 °C for 5 min. The PCR products were then separated on a 1.5% agarose gel and visualized under a ChemiDoc XRS gel photo documentation system (Bio-rad, Hercules, CA, USA). The intensity of each band was determined by ImageJ software.

**Table 1:** Nucleotide sequences used for RT-PCR detection of cytokine mRNA expression

Gene	Primers	Product size
IL-8	Forward primer 5′-TCC AAA CCT TTC CAC CCC AA-3′ Reverse primer 5′-ACT TCT CCA CAA CCC TCT GC-3′	153 bp
GAPDH	Forward primer 5’-CTG ACT TCA ACA GCG ACA CC-3′ Reverse primer 5′-GTG GTC CAG GGG TCT TAC TC-3′	172 bp

### Monocyte migration assay

Transwells containing 5-μm pore size polycarbonate filters (Corning, NY, USA) were precoated with human fibronectin by incubation at 37 °C for 1 h and washed twice in PBS. The transwells were inserted into the lower chambers, which contained starvation medium (RPMI 1640 containing 0.1% (v/v) fetal bovine serum) alone for the negative control or medium supplemented with either 10 ng/ml platelet-derived growth factor-BB (PDGF-BB) for the positive control or 10–10,000 ng/ml sHP for the infection groups. THP-1 cells were cultured in starvation medium for 2 d before testing. Cells were centrifuged at 200 g for 5 min, resuspended in 1 ml starvation medium and passed through a 40 μm cell strainer (BD Falcon, Bedford, MA, USA). THP-1 cells (10^5^ cells) and CEAE-KP (0, 16 or 32 μg/ml) were added to the upper chambers, incubated at 37 °C for 1.5 h and washed twice with PBS. Next, 10% (w/v) formalin was added and incubated at room temperature for 10 min to fix the cells. Cells were stained with Cytoquick (Muto Pure Chemical, Japan), gently washed with water to remove the excess stain, and dried overnight. Cells that did not migrate were wiped away with a damp cotton swab. The migrated cells were viewed in 10 fields of view under a light microscope with a 20× objective lens, and the cells were counted using ImageJ software.

### Neutrophil migration assay

Each 5-μm Transwell was precoated with human fibronectin and inserted into the lower chamber, which contained starvation medium alone for the negative control or medium supplemented with 10 ng/ml IL-8 for the positive control. HL-60 cells were differentiated into neutrophils by induction with 1.25% (v/v) DMSO for 6 d, and then DMSO-differentiated HL-60 cells (10^5^) and CEAE-KP (0, 16 or 32 μg/ml) were added into the upper chambers and incubated at 37 °C for 30 min. The cells on the membrane were then washed, fixed, stained and counted as described in the monocyte migration method.

### Statistical analysis

The data are presented as the mean ± one standard deviation (SD). Statistical analysis was performed with GraphPad Prism 6 (GraphPad Software, San Diego, CA, USA). One-way ANOVA followed by Bonferroni post-tests was used to compare the control and test groups. The effects of the treatment over time were analyzed by two-way ANOVA followed by Bonferroni post-tests. Values of *p* ≤ 0.05 were considered statistically significant.

## Results

### Cytotoxicity of CEAE-KP in the AGS cell line

To evaluate the toxicity of CEAE-KP and select an acceptable (non-toxic) concentration and incubation time, the MTT assay was performed. According to our previous study, the MIC of CEAE-KP was 32 μg/ml [29], and concentrations of CEAE-KP ranging from 8 to 128 μg/ml were examined for cytotoxicity against AGS cells. AGS cell viability was reduced by CEAE-KP in a concentration- and time-dependent manner (Table 2); CEAE-KP at concentrations of 64 and 128 μg/ml significantly reduced AGS cell viability to 69.3 ± 7.1% and 36.1 ± 3.7% at 6 h, 56.7 ± 9.8% and 10.8 ± 5.4% at 12 h, and 16.6 ± 4.4% and 1.2 ± 0.6% at 24 h, respectively. In addition, CEAE-KP at 32 μg/ml was cytotoxic at the longest incubation time (24 h), reducing cell viability to 72.8 ± 6.6%. However, 8 and 16 μg/ml CEAE-KP was not toxic to the AGS cell line at any assayed time point. In a previous study, the ethanol extract of KP decreased HL-60 cell viability in a dose- and time-dependent manner, and no cytotoxic effect was noted at concentrations of 0–20 μg/ml for 24 h [30]. Thus, subsequent experiments used CEAE-KP at 8 or 16 μg/ml with an incubation time that was limited to 24 h.

**Table 2:** Cytotoxicity of CEAE-KP on AGS cells by MTT assay

Treatment	Cell viability (%)		
	Time		
	6 h	12 h	24 h
Media	100.00 ± 3.47	98.29 ± 3.15	98.67 ± 2.44
DMSO	105.00 ± 10.80	91.56 ± 7.71	92.98 ± 6.37
CEAE-KP 8 μg/ml	96.69 ± 15.36	91.25 ± 5.51	91.16 ± 9.64
CEAE-KP 16 μg/ml	88.08 ± 12.90	87.65 ± 4.36	92.44 ± 4.19
CEAE-KP 32 μg/ml	90.80 ± 13.78	90.36 ± 5.09	72.76 ± 6.62^***^
CEAE-KP 64 μg/ml	69.31 ± 7.05^***^	56.74 ± 9.78^***^	16.57 ± 4.43^***^
CEAE-KP 128 μg/ml	36.10 ± 3.68%^***^	10.79 ± 5.40^***^	1.22 ± 0.58^***^

The results are presented as the mean ± SD of three independent experiments. Statistical analysis was performed using two-way ANOVA with Bonferroni post hoc tests. The symbol (***) indicates a *p*-value ≤0.001 compared to the media control

### Inhibition by CEAE-KP of *H. pylori*-induced IL-8 production and IL-8 mRNA expression

**Fig. 1:**
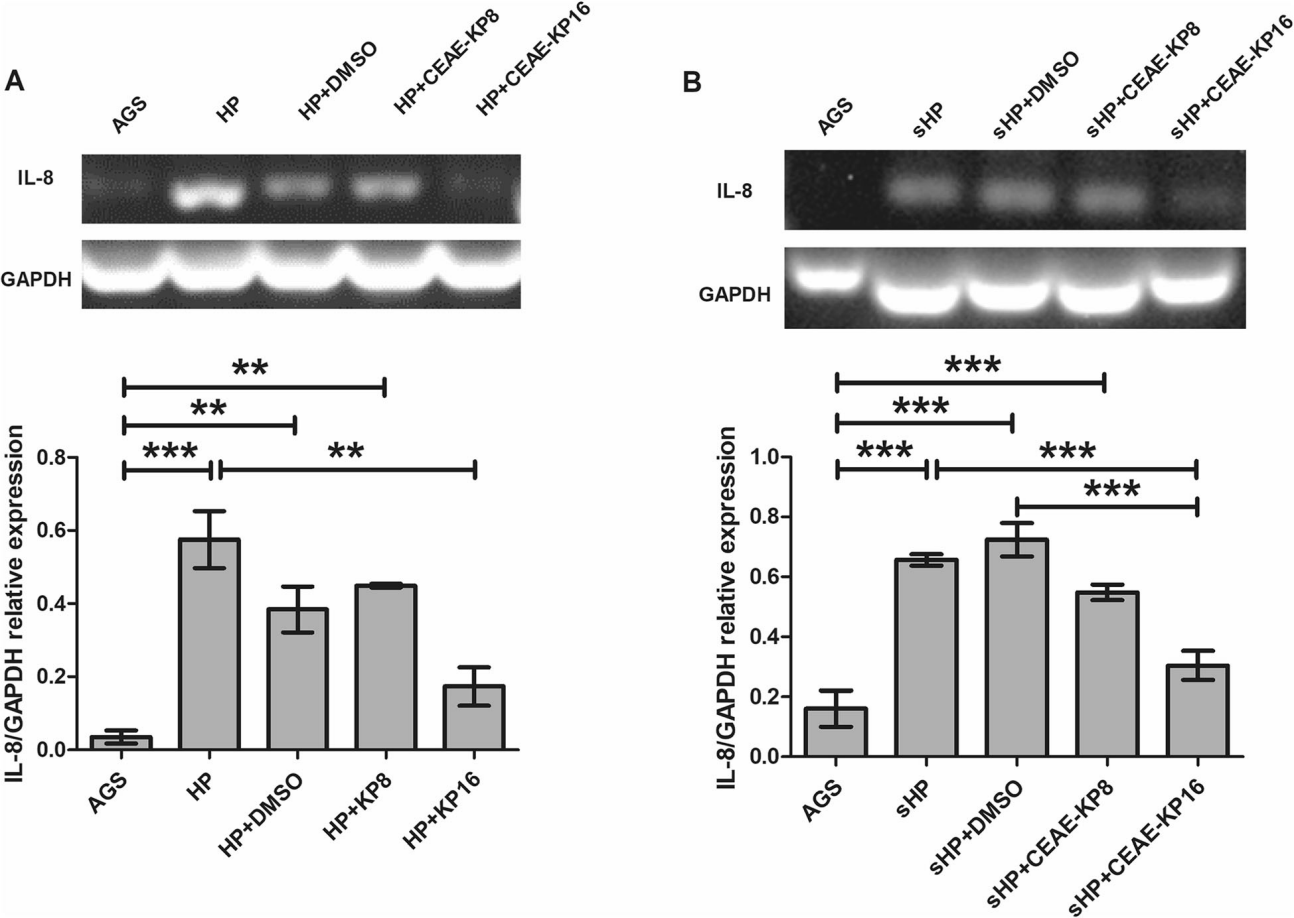
Effect of CEAE-KP on IL-8 mRNA expression in AGS cells induced by *H. pylori***(a)** or sHP **(b)** at 12 h by RT-PCR. DMSO was used as a vehicle control. Representative RT-PCR agarose gel electrophoresis images are shown. IL-8 mRNA expression was determined relative to GAPDH. The results are presented as the mean ± SD of three independent experiments. Statistical analysis was performed using one-way ANOVA with Bonferroni post hoc tests. The symbols (**) and (***) indicate *p*-values ≤0.01 and ≤ 0.001, respectively

IL-8 is one of the most responsive proinflammatory cytokines in *H. pylori* infection. AGS cells produced IL-8 after in vitro infection with *H. pylori* in a time-dependent manner (Table 3), with the highest IL-8 level detected at 24 h (421.3 ± 57.3 pg/ml). A low CEAE-KP concentration (8 μg/ml) did not significantly decrease the IL-8 level at any assayed incubation time, but 16 μg/ml CEAE-KP significantly reduced the IL-8 level by 2.3-fold at 6 h and by 2.07-fold at 12 h. However, the inhibitory effect of CEAE-KP on IL-8 secretion was limited to up to 12 h, with no significant IL-8 reduction after incubation for 24 h. Both *H. pylori* and sHP dramatically induced IL-8 mRNA expression, and the increased IL-8 was significantly downregulated by CEAE-KP at 16 μg/ml (Fig. 1a and b).

**Table 3:** Effect of CEAE-KP on IL-8 secretion by AGS cells infected with *H. pylori*, as measured by ELISA

Treatment	IL-8 (pg/ml)		
	Time		
	6 h	12 h	24 h
Media	0.064 ± 0.04	0.00 ± 0.01	5.17 ± 6.36
*H. pylori*	133.10 ± 59.75	149.30 ± 18.39	421.30 ± 57.30
*H. pylori +* DMSO	123.20 ± 18.66	147.60 ± 24.24	429.80 ± 31.03
CEAE-KP 8 μg/ml	138.10 ± 40.57	161.50 ± 19.22	499.30 ± 22.80
CEAE-KP 16 μg/ml	57.68 ± 18.92^**^	72.15 ± 18.64^**^	410.20 ± 30.46

The results are presented as the mean ± SD of three independent experiments. Statistical analysis was performed using two-way ANOVA with Bonferroni post hoc tests. The symbol (**) indicates a *p*-value ≤0.01 compared to the *H. pylori-*infected control

### Induction of monocyte chemotaxis by sHP

**Fig. 2:**
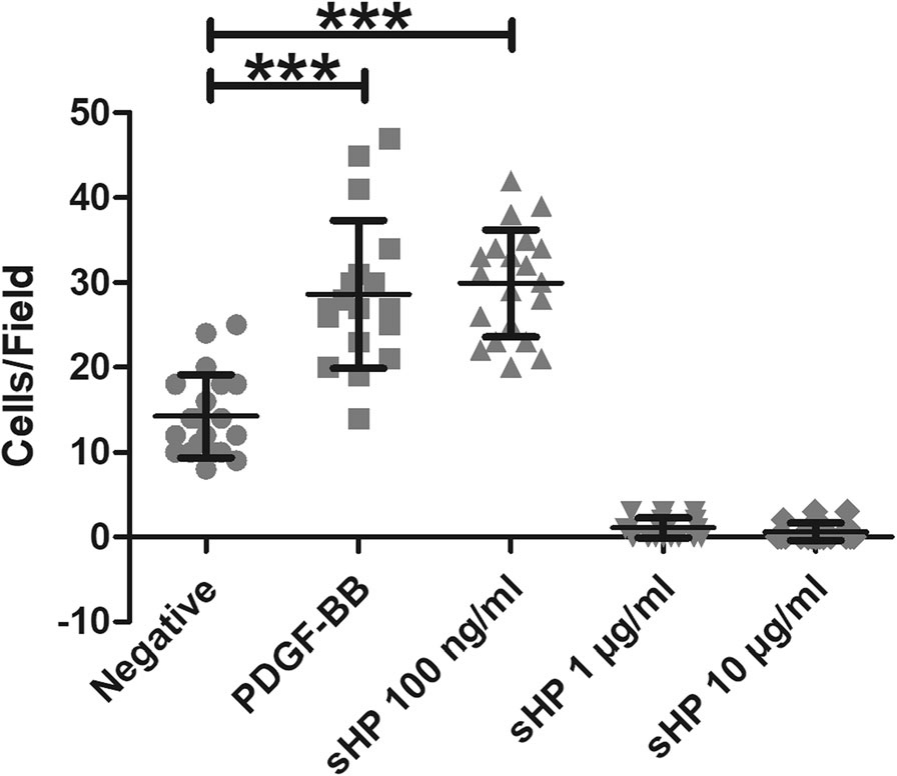
Effect of sHP (0–10 μg/ml) on the chemotaxis of THP-1 cells. PDGF-BB (10 ng/ml) was used as a positive control, and DMSO was used as a vehicle control. The results are presented as the mean ± SD of three independent experiments. Statistical analysis was performed using one-way ANOVA with Bonferroni post hoc tests. The symbol (***) indicates a *p*-value ≤0.001

To investigate the optimal concentration of sHP to activate chemotaxis in THP-1 cells, sHP was added to the lower chamber at concentrations of 0.1–10 μg/ml. At 100 ng/ml, sHP induced chemotaxis in 30 ± 6 cells/10 fields of view, which was similar to that of PDGF-BB (28 ± 8 cells/10 fields of view). Concentrations of sHP greater than 1 μg/ml rapidly attracted THP-1 cells into the lower chamber, leaving no cells on the transmembrane (Fig. 2). These results demonstrated that some intracellular *H. pylori* proteins serve as chemoattractants for monocytes, while sHP at a concentration of 100 ng/ml was optimal under this assay condition for further experiments.

### Inhibition by CEAE-KP of monocyte chemotaxis induced by sHP

**Fig. 3:**
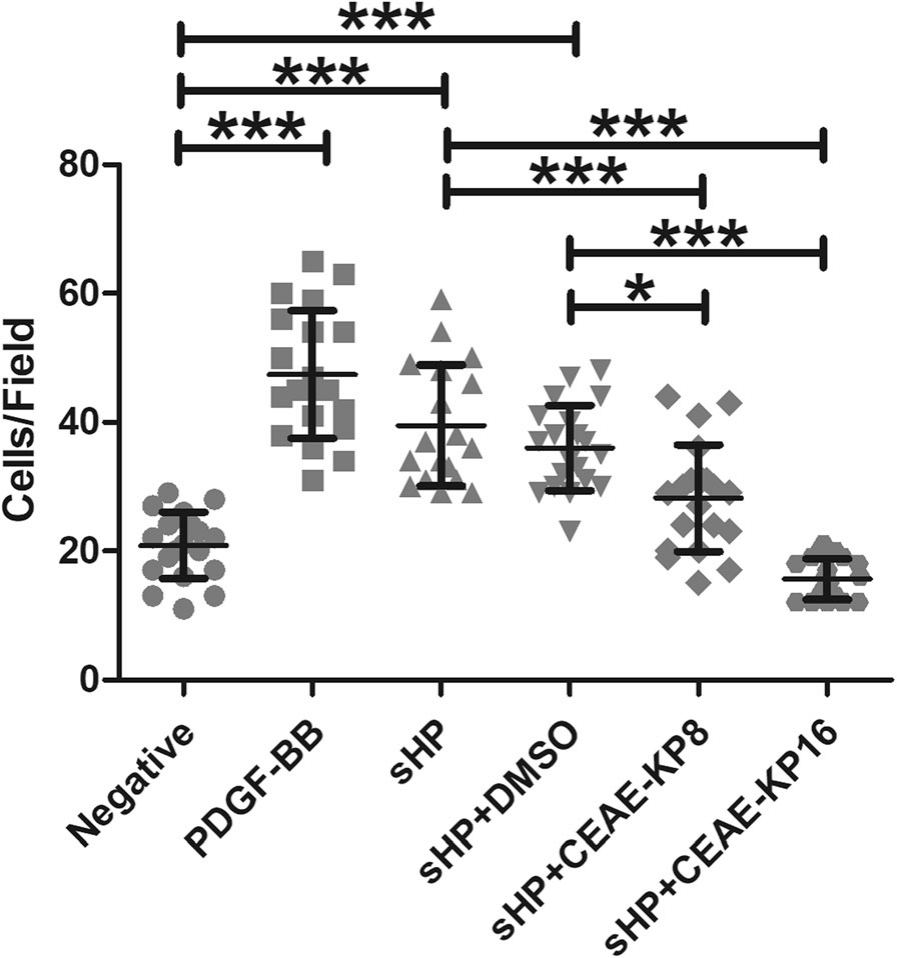
Effect of CEAE-KP on THP-1 cell chemotaxis induced by 100 ng/ml sHP. PDGF-BB (10 ng/ml) was used as the positive control, and DMSO was used as the vehicle control. The results are presented as the mean ± SD of three independent experiments. Statistical analysis was performed using one-way ANOVA with Bonferroni post hoc tests. The symbols (*) and (***) indicate *p*-values ≤0.05 and ≤ 0.001, respectively

To study the effect of CEAE-KP on the chemotaxis of THP-1 cells, CEAE-KP and THP-1 cells were cocultured in the upper chamber. The number of chemotactic cells was significantly reduced (1.4-fold) by CEAE-KP at 8 μg/ml and further reduced by 2.5-fold at 16 μg/ml (Fig. 3).

### Inhibition by CEAE-KP of IL-8-induced neutrophil chemotaxis

**Fig. 4:**
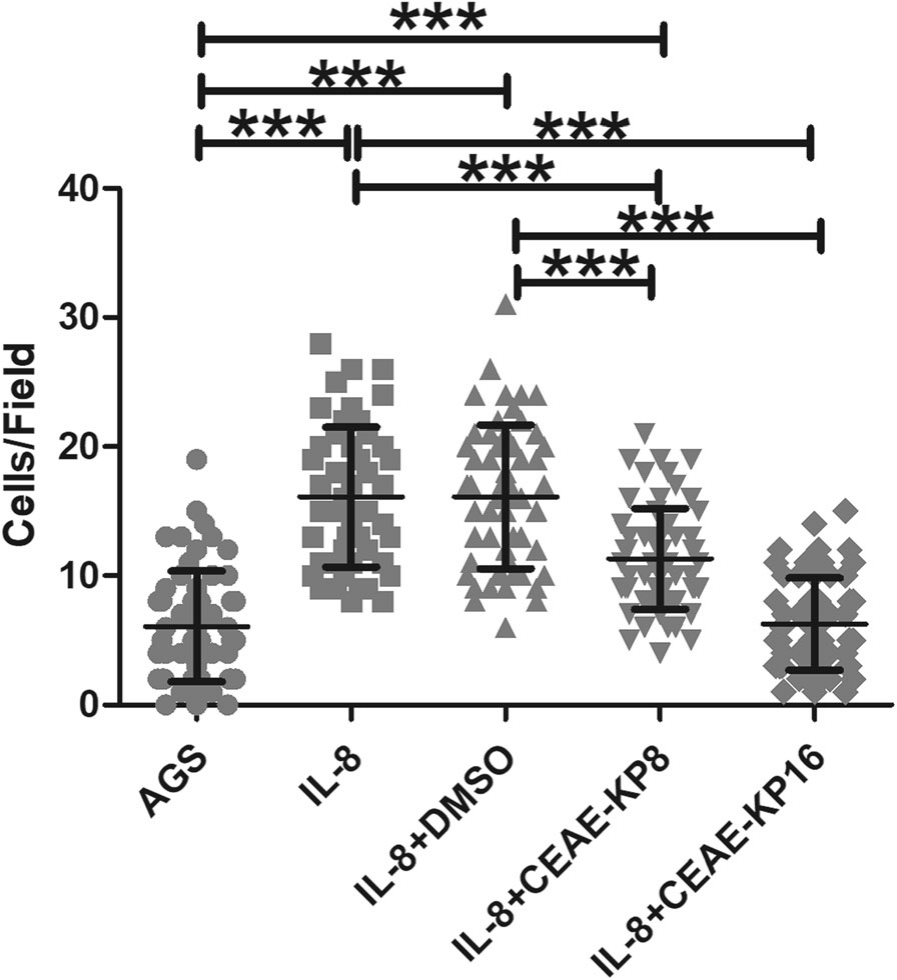
Effect of CEAE-KP on differentiated HL-60 cell chemotaxis induced by IL-8. IL-8 (10 ng/ml) was used to attract the cells and as a positive control, while DMSO was used as a vehicle control. The results are presented as the mean ± SD of three independent experiments. Statistical analysis was performed using one-way ANOVA with Bonferroni post hoc tests. The symbol (***) indicates a *p*-value ≤0.001

Neutrophil participation in gastric inflammation is related to the clearance of *H. pylori*. Neutrophil migration is highly responsive to IL-8; therefore, IL-8 secreted by gastric epithelial cells is likely to be an important mediator that induces neutrophil migration to the sites of infection. To study the effect of CEAE-KP on neutrophil chemotaxis to IL-8, CEAE-KP and differentiated HL-60 cells were cocultured in the upper chamber. The number of chemotactic cells was significantly reduced (1.4-fold) by CEAE-KP at 8 μg/ml and further reduced by 2.5-fold at 16 μg/ml (Fig. 4).

## Discussion

Most *H. pylori*-infected people are asymptomatic, with only slight gastric inflammation. The asymptomatic nature of this disease results in delayed treatment and can subsequently lead to enhanced inflammation that can affect the severity and progression of gastric disease, starting with gastritis and progressing through atrophy, metaplasia, dysplasia and gastric cancer [31]. Severe stages of gastritis (gastritis stages III and IV) are associated with neoplastic prevalence and *H. pylori* infection status [32]. Unfortunately, resistance to clarithromycin, amoxicillin and metronidazole is increasing in *H. pylori,* which reduces the efficacy of triple therapy eradication [33]. Consequently, the Toronto consensus recommended a bismuth quadruple regime as a first-line therapy to improve the worldwide *H. pylori* eradication rate [34]. However, bismuth is a heavy metal that is toxic to mammalian cells [35]. For example, human embryonic kidney cells exposed to bismuth for 48 h showed a decreased survival rate, and autophagy was detected [36]. Hence, medicinal plants that exhibit antibacterial, anti-inflammatory, antiulcer, and antiapoptotic effects are useful alternative approaches to eradicate *H. pylori* infection and gastric cancer treatment. Several active compounds in herbal plants have been demonstrated to have pharmacological and anti-*H. pylori* activities, such as curcumin, quercetin, allicin, geraniol, dimethoxyflavone, crocin and safranal [37–40]. Anti-inflammatory action focusing on reducing IL-8 has been demonstrated in many natural products, including *Ixeris chinensis* [41], *Sanguinaria canadensis* [42], *Lotus tetragonolobus* and *Maackia amurensis* [43].

*K. parviflora*, a plant in the ginger (*Zingiberaceae*) family, flourishes in the Asia-Pacific region and has been used as a traditional medicine by some ethnic groups in Thailand to treat digestive tract disorders for over a century [44]. In a previous study, CEAE-KP was found to be the most effective extract in terms of active ingredients that disrupt *H. pylori* internalization and growth (MIC = 32 μg/ml) [26, 29]. However, due to the potential safety concern of KP extracts, the in vitro cytotoxic effect of CEAE-KP on AGS cells was evaluated in this study. CEAE-KP showed time- and concentration-dependent cytotoxicity at concentrations of 32 μg/ml and above but not at low concentrations (8–16 μg/ml) for up to 24 h (the longest time point assayed), with over 90% cell survival relative to the control.

According to a genome-wide expression microarray, IL-8 was the single most upregulated gene in AGS cells after stimulation with *H. pylori* [45]. Thus, IL-8 was used as a marker of gastric cell inflammation from *H. pylori* infection. The maximum noncytotoxic concentration of CEAE-KP (16 μg/ml) reduced IL-8 secretion from infected AGS cells by approximately 60% at 6 and 12 h, but this then declined, and no inhibition of IL-8 secretion was observed at 24 h. Similarly, the mRNA level of IL-8 was decreased by CEAE-KP treatment. These results indicated that CEAE-KP has an inhibitory effect that downregulates proinflammatory cytokines induced by *H. pylori* infection. The highest DMSO concentration (0.016% (v/v)) in CEAE-KP was also tested in this study as a vehicle control and showed no significant effect on IL-8 secretion in AGS cells. Hollebeeck et al. found that DMSO ranging from 0.05–1% did not have any significant effect on IL-8 secretion in intestinal Caco-2 cells. However, DMSO at 0.1 and 0.5% downregulated IL-1, IL-6 and COX-2 mRNA expression [46].

Neutrophils are IL-8-sensitive inflammatory cells that have high expression of IL-8 receptors (CXCR1 and CXCR2) on their membrane [47, 48]. Ligand activation of the receptor induces signaling molecules in the neutrophil chemotaxis cascade, such as phosphoinositide 3-kinase-γ, phospholipase C and extracellular signal-regulated kinase. Consequently, neutrophils undergo chemotaxis towards the infection site, which is an IL-8 source [49]. The extensive IL-8 production induced by *H. pylori* infection of AGS cells (a gastric adenocarcinoma cell line) was reduced by the presence of CEAE-KP, which also decreased IL-8-induced DMSO-differentiated HL-60 cell (in vitro neutrophil model) chemotaxis by 21.4–57.1%. Hence, CEAE-KP downregulates both IL-8 expression and its further inflammatory mechanism in neutrophil chemotaxis.

Macrophages play a pivotal role in the innate immune response by eliminating microbes by phagocytosis. However, monocyte chemotaxis occurs before these cells differentiate into macrophages and infiltrate across the endothelial cells to the infected tissue. Chemokines or bacterial antigens act as chemotactic agents and are required to activate monocytes. Monocyte chemotaxis is induced by *H. pylori* proteins through the N-formyl peptide receptor family (FPR) signaling pathway [50, 51]. The induction of THP-1 cell (in vitro monocyte model) chemotaxis by sHP was confirmed in this study, and sHP activated a comparable number of migrated cells as PDGF-BB, a well-known monocyte chemoattractant [52]. Nevertheless, monocyte chemoattraction to sHP was 10-fold lower than that of PDGF-BB, based upon their respective optimal concentration. Some natural products exhibit monocyte attraction, while isothiocyanate decreased stromal cell-derived factor-1α (SDF-1α)-mediated monocyte chemotaxis and phagocytosis through inhibition of the NF-kB and mitogen-activated protein kinase (MAPK) pathways [53]. CEAE-KP has previously been shown to inhibit monocyte-endothelial adhesion [54], which is the mandatory step before monocyte infiltration into the surrounding tissue. However, this study is the first to report that CEAE-KP downregulated monocyte activity at an earlier step and reduced sHP-mediated monocyte chemotaxis by 30–60%.

The inhibition of IL-8 production and leukocyte chemotaxis is probably driven by active polymethoxyflavones in the CEAE-KP. HPLC analysis of the ethyl acetate fraction of KP showed 12 main peaks of 7-methoxyflavones composed of 4′-hydroxy-5,7-dimethoxyflavone; 5,7,3′,4′- tetramethoxyflavone; 3,5,7,3′,4′ –pentamethoxyflavone; 5,7-dimethoxyflavone; 5,7,4′-trimethoxyflavone; 3,5,7-trimethoxyflavone; 3,5,7,4′-tetramethoxyflavone; 5-hydroxy-3,7,3′,4′-tetramethoxyflavone; 5-hydroxy-7-methoxyflavone; 5-hydroxy-7,4′-dimethoxyflavone; 5-hydroxy-3,7-dimethoxyflavone; and 5-hydroxy-3,7,4′-trimethoxyflavone [55]. Dimethoxyflavone and trimethoxyflavone have been indicated as major active compounds that suppress proinflammatory cytokines (induced by IL-1β or TNF-α with IL-17A) via the p38/STAT1 and STAT3 pathways. In addition, a mixture of major active compounds and crude extract of KP more strongly suppressed TNF-α and MMP-13 mRNA expression than individual compounds [54, 56]. The major component 5,7,4′-trimethoxyflavone also exhibits antiplasmodial, antifungal and antimycobacterial activities [57].

Thus, a comparative study of KP crude extracts and pure active compounds (both individually and as mixtures) should be performed to determine their effect on chemotaxis.

### Conclusion

CEAE-KP possesses multifunctional anti-inflammatory activity against *H. pylori* infection by regulating several major inflammatory mechanisms. CEAE-KP inhibited both the initial inflammatory signal (IL-8) from *H. pylori*-infected AGS cells (human gastric cell model) and the relevant leukocyte recruitment. Taken together, KP might be suitable as a part of a novel alternative regimen for ameliorating the inflammation mediated by *H. pylori* infection and decreasing the severity of the disease.

## Data Availability

All data generated or analysed during this study are included in this published article.

## Abbreviations

KP: *Kaempferia parviflora*
CEAE: Crude ethyl acetate extract;
sHP: *H. pylori* soluble protein
IL: Interleukin
DMSO: Dimethyl sulfoxide
PDGF-BB: Platelet-derived growth factor-BB
